# Diagnosis and Treatment of Carpal Tunnel Syndrome in A Tertiary Care Center in Mexico City

**DOI:** 10.29252/wjps.9.2.219

**Published:** 2020-05

**Authors:** I Campos-Serna, Jaime Aron Garcia-Espinoza, Alberto Ignacio Cahuana-Quispe, Cuahutemoc Marquez-Espriella, Marco Antonio Cuervo-Vergara, Jose Carlos Martinez-Lopez, Rodrigo Dávila-Diaz

**Affiliations:** Division of Plastic and Reconstructive Surgery, Hospital Central Sur de Alta Especialidad de Petróleos Mexicanos, Mexico City, Mexico

**Keywords:** Carpal tunnel syndrome, Phalen, Tinel, Neuropathy

## Abstract

**BACKGROUND:**

Carpal tunnel syndrome is the most common peripheral neuropathy affecting patients at productive age and has an important economical impact on those who suffer it. This study assessed the diagnostic performance of carpal tunnel syndrome´s signs and described the epidemiology at a tertiary care center in Mexico City.

**METHODS:**

All patients diagnosed with carpal tunnel syndrome during a five-year period were included. Demographic data, electromyography results, positive clinical signs and the severity score according to the Italian scale were recorded. Diagnostic accuracy of Tinel and Phalen´s signs were calculated via odds ratio.

**RESULTS:**

Totally, 650 patients were diagnosed and treated during a five-year period, 84% were female and 16% male, and the mean age was 55.8 years. The associated comorbidities were trigger finger (36.1%), thyroid disease (25.6%) and diabetes (20%). Diagnosis yielded for Phalen and Tinel signs were variable in each of the study groups (males and females) and showed to be beneficial in diagnosis of the disease.

**CONCLUSION:**

Carpal tunnel syndrome is a complex disease in which clinical signs remain the cornerstone of diagnosis. Extension studies are useful to assess the severity of the disease.

## INTRODUCTION

Carpal tunnel syndrome is the most frequent peripheral mononeuropathy worldwide with an estimated prevalence of 1 to 5% and an incidence of 2.2 to 5.4% per 1000 people.^1^ Although its pathogenesis is multifactorial, a higher prevalence and incidence has been found in women because they have a narrower carpal tunnel prone to higher internal pressures that cause neural compression.^2^ Identified risk factors for this disease are obesity, female gender^3^ and comorbidities such as diabetes mellitus [Odds Ratio (OR): 2.2],^4^ hypothyroidism (OR: 1.4),^5^ rheumatoid arthritis (OR: 2.2)^6^ and the use of drugs such as Anastrazole (OR: 2.6).^7^

Other factors associated are vicious positions of the wrist and repetitive activities with the upper extremities, although a statistical relation has not been proven. Neural damage in carpal tunnel syndrome is developed due to the increased pressure inside the tunnel. It disrupts axonal transport and compromises perineural irrigation causing ischemia of the median nerve. This phenomenon has physical and psychological implications. It mostly affects patients in their productive years of life, thus representing significant economical losses.^8^

Despite medical advances, the gold standard for diagnosis is a thorough clinical history and physical exam. The classic syndrome is described with intermittent paresthesias of the middle and ring finger, it mostly occurs at night, as well as dysesthesias that wakes the patient up.^9^^,^^10^ Severe manifestations such as loss of sensation and muscle atrophy of the thenar region, appear later in the course of the disease. In the physical examination, pain on palpation is often found. Tinel and Phalen maneuvers increase internal pressure in the tunnel evoking paresthesia in the territory of the median nerve, with a sensibility of 42-85% for Phalen sign and 38-100% for Tinel sign, and a specificity of 54-98% and 55-100%, respectively.^11^

Diagnostic tools are not needed for the diagnosis, they only assist confirming it. Yet, electromyography (EMG) allows to rule out other causes of neural damage and establishes the degree of axonal injury in a systematic and concise way.^12^ Other tools such as wrist ultrasound have an acceptable diagnostic performance, but require specialized equipment and medical staff that results in higher costs.^13^ Treatment continues to be an important point of study, conservative strategies such as postural changes or medical treatment with gabapentin have failed to show completely effective in the resolution of symptoms in the short and long term.^14^

Surgical release of the transverse ligament of the carpal tunnel is the treatment of choice for most cases, it has proven to stop axonal degeneration and has a therapeutic effect in the long term (up to two years) compared with the use of local steroids. Surgical treatment includes the conventional approach with an open incision in the volar aspect of the hand, and the endoscopic approach with a minimally invasive incision trough which a blade is inserted to release the ligament under direct vision.^15^

Studies show the latter to be less painful and to have a shorter recovery time. Yet, it requires highly expensive and specialized equipment, as well as experienced staff in order to be safe and effective.^16^ The research and teaching ethics committee of our institution approved the study with number 46/19HCSAE and did not apply the informed consent of patients, since it was a retrospective study where the identity of the patients was protected. This study was undertaken to describe the epidemiological profile of the population with diagnosis of carpal tunnel syndrome treated at a tertiary care center in Mexico City (age, gender, affected side, risk factors, comorbidities, EMG results). Also, the sensibility, specificity, positive predictive value (PPV) and negative predictive value (NPV), a probability of occurrence for Odds Ratio (OR) among clinical signs and electrical conduction studies, as well as the diagnostic accuracy of clinical signs (Phalen, Tinel and Durkan) were determined.

## MATERIALS AND METHODS

The study includes patients with diagnosis of carpal tunnel syndrome treated in “Hospital Central Sur de Petroleos Mexicanos” in Mexico City, Mexicoa during 5-year period. Epidemiologic variables such as age, gender, affected side, bilaterality, sensibility, specificity, PPV and NPV, diagnostic accuracy and OR of clinical signs were compared with electric conduction studies. Severity of disease, history of local steroids, physical therapy, comorbidities, length of symptoms, number of procedures, recovery time, postoperative complications and need of reintervention were also described.

## RESULTS

Totally, 650 patients treated during five years in our unit were included, 84% were female and 16% male, and the average age was 55.8 years, with a range of 48 to 63 years. The evolution of the disease had an average of 5.86 months with a range of 3 to 8 months. Our population presented concomitant comorbidities as trigger finger (36.1%), thyroid diseases (25.6%) and diabetes (20%). The patients were divided in two groups according to gender, as female group having an average age of 55.1 years with a range of 45.2 to 62.7 years and an average evolution of 5.8 months with a range of 3 to 7 months. Likewise, 61% of these patients underwent surgical treatment and had a mean postoperative recovery of 12 days with a range of 11 to 14 days, and unilateral condition was found in 61% and bilateral in 39% of cases. 

In the male group, the average age was 59.27 years old with a range of 52 to 65 years. They presented an evolution of 7.57 months with a range of 4.5 to 9 months, 52% of these patients underwent surgical treatment and had a 13-day mean postoperative recovery with a range of 8 to 14 days. Unilateral disease was present in 62% and bilateral in 38%. All of the patients underwent electromyographic examination and was compared with the Italian clinical classification. 

**Fig. 1:**
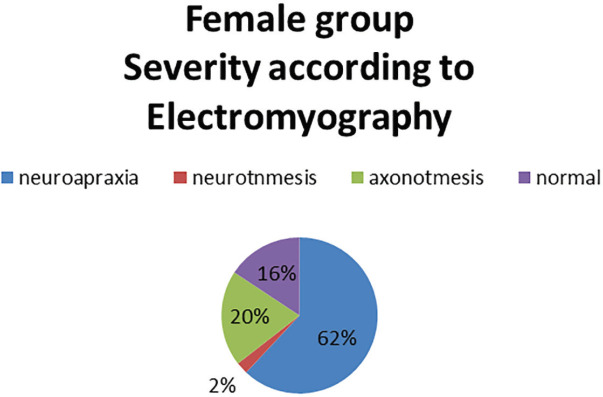
Severity according to electromyographyc test

**Fig. 2:**
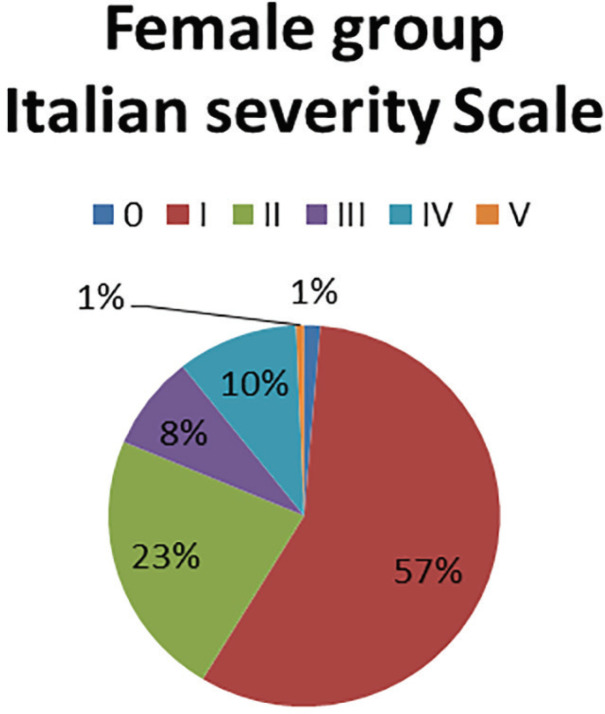
Severity according to Italian scale in female group

**Fig. 3:**
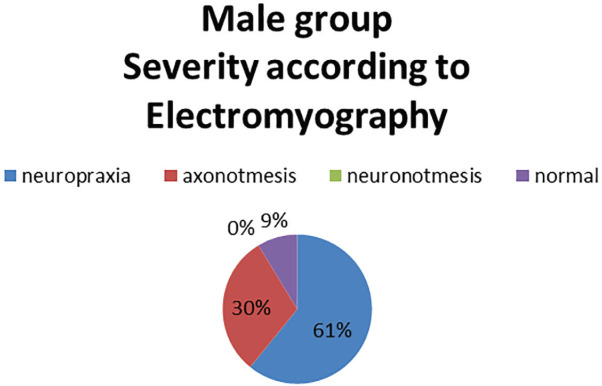
Severity according to electromyographyc test

In the Female group it was found that 62% had the electrodiagnostic of neuropraxia and some cases were reported with neurotmesis (Figure 1). When compared with the Italian clinical classification, it was found that the vast majority presented clinical data compatible with incipient degrees of the disease (Figure 2). In the male group, it was observed that 61% had an electrodiagnostic of neuropraxia, 30% axonotmesis and no cases of neurotmesis (Figure 3). 

**Fig. 4:**
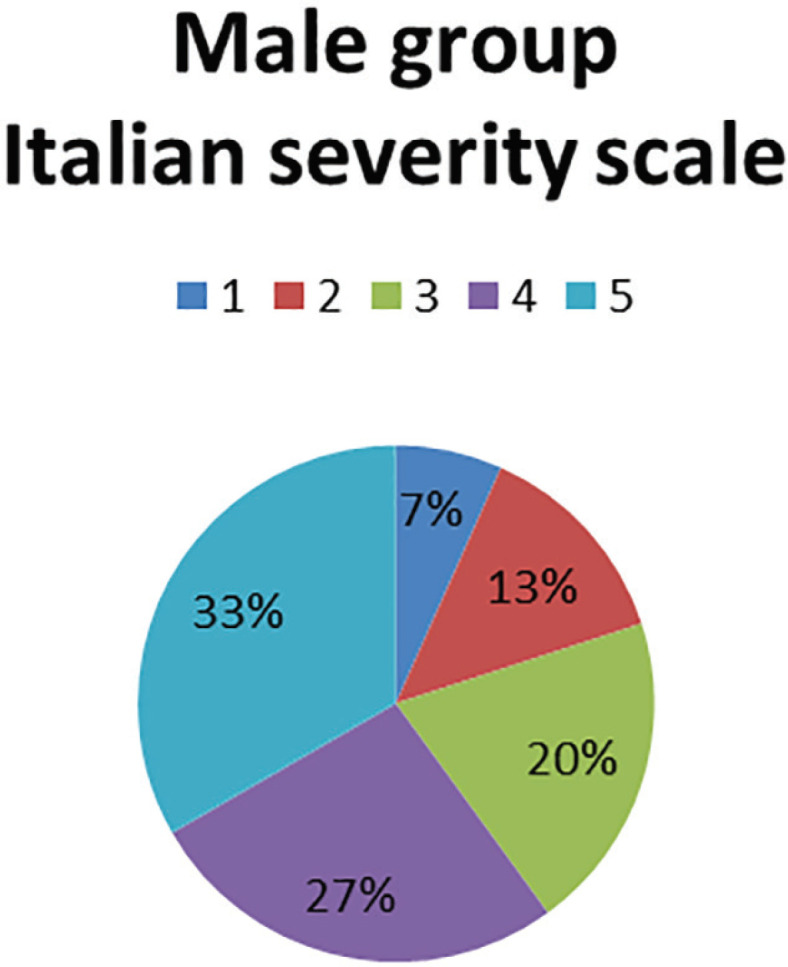
Severity according to Italian scale in male group

When measuring the severity of the disease with the Italian clinical classification, more than 50% presented grade IV and V of severity. However, when trying to correlate both ways of measuring the severity of the disease (clinical and electromyography), there was not any correlation between groups. When we compared diagnostic performance of the signs with electrodiagnosis; in the female group, we found a sensitivity of 72.8, specificity of 43.7, PPV of 89.2, NPV of 73.6, an OR of 2.9 and a diagnostic accuracy test of 0.6 (Figure 4). 

For the Phalen sign, a sensitivity of 69, specificity of 44.4, PPV of 89.9, negative predictive value of 84.4, an OR of 2.8 and a diagnostic accuracy test of 0.67. In the male group, we found a sensitivity of 66.6, specificity of 50, PPV of 87.5, NPV of 77.7, OR of 2 and a diagnostic accuracy test of 0.56. For the Phalen sign we found a sensitivity of 65, specificity of 50, positive predictive value 92.8, NPV of 86.9, an OR of 3.7 and a diagnostic accuracy test of 0.6.

## DISCUSSION

Carpal tunnel syndrome is one of the most frequent complaints for a hand surgeon. Functional prognosis of the hand is based on the median nerve compression chronicity; that´s why clinical data is crucial for an early detection and treatment. Our study showed similar results observed in other series regarding female gender predominance as a result of a narrower carpal tunnel.^17^ Mack *et al.* in their series reported an incidence of 74%^18^ and Destefano *et al.* reported a 63% female predominance, respectively; when compared to our findings of 84%. Regarding age range, they also obtained similar results demonstrating that this pathology affected the productive ages of life and impacted the economy of society.^19^

In our study, the evolution of the condition from the onset of symptoms to surgery had an average of 5.86 months with a range of 3 to 8 months. Our population presented as comorbidities, thyroid diseases (25.6%) and diabetes mellitus (20%), in contrast to Destefano´s series that showed only 2 and 5%, respectively. In our population, there was a particular association of trigger finger in the same neuropathic hand in 36.1%, which led us to think of a probable pathological association between different pathologies that afflicted the hand, so it is necessary to conduct studies that allow us to determine the strength of association between these pathologies and which of them could be the trigger.^19^

We decided to make two groups according to gender, because there are currently no other studies that allow us to identify differences regarding age in the natural history of this pathology. Our results are similar to other researchers,^18^ however, we found differences in the early onset of symptoms in women and a greater need for surgery in less evolution time (Table 1). Electrodiagnostic study is an adjuvant tool and not all patients need its performance; since a good medical history and physical examination is enough to make diagnosis and to start treatment. Electrodiagostic assessment is needed for staging and supporting a work disability (in active workers). 

**Table 1:** Epidemiological and demographic variables

**Variable**		**Female** **(84%)**	**Male** **(16%)**
Age	Mean	55.1	59.2
	IQR	45.2-62.7 years	52-65 years
Time of evolution	Mean	5.8	7.5
	RIC	3-7 months	4.5-9 months
Surgical treatment		61%	52%
Recovery time	Mean	12 days	13 days
	IQR	11-14 days	8-14 days
Laterality	Unilateral	61%	62%
	Bilateral	39%	38%

IQR: intercuartile range

Neuropraxia was found in 62% and 61%, respectively and it contrasted with the Italian clinical classification, in which women presented incipient stages and men severe stages; however, at the time of the clinical-electrodiagnostic correlation, they showed no statistical significance between groups. In a systematic review, sensitivities ranging from 42% to 85% were illustrated for the Phalen sign and 38% to 100% for the Tinel sign and the specificity ranged from 54% to 98% and 55 to 100%, respectively.^11^

The results obtained in this study demonstrated weak sensitivity and specificity measurements; however, the calculation of the predictive values in each of them demonstrated utility to confirm the diagnosis in a clinical ground, in the same way the OR calculations allowed us to establish the use of both clinical signs for diagnosis.^11^ What we observed when analyzing the data was a greater diagnostic performance of the Tinel sign for the women´s group and a greater diagnostic performance of the Phalen sign for men (Table 2). 

**Table 2:** Diagnostic performance of clinical signs by group

**Variable**		**Female**	**Male **
Tinel sign	Sensibility	72.8	66.6
	Specificity	43.7	50
	Positive predictive value	89.2	87.5
	Negative predictive value	73.6	77.7
	Likelihood of occurrence (OR)	2.9	2
	Diagnostic accuracy test	0.6	0.56
Phalen sign	Sensibility	69	65
	Specificity	44.4	50
	Positive predictive value	89.9	92.8
	Negative predictive value	84.4	86.9
	Likelihood of occurrence (OR)	2.8	3.7
	Diagnostic accuracy test	0.67	0.6

Carpal tunnel syndrome continues to be the most frequent compression neuropathy, with the female gender being the most affected. The highest incidence peak affects productive ages despite gender, so an early diagnosis is essential to reduce institutional costs that condition this pathology. The clinical signs of Tinel and Phalen remain fundamental elements of the diagnosis of the disease, despite their variable diagnostic performance, they allow to establish the existence of neural compression, when used together. 

Electromyography is used to establish the neural damage type that exists. Despite reports of high diagnostic performance of the Durkan sign, in our group it was not explored, leaving a line of research to determine its effectiveness in a Latin American population. For this reason, new lines of research should be created to establish associations with other hand pathologies, effective screening tests, medical care protocols in specialized centers and surgical techniques that help reducing the days of disability caused by the surgical resolution of this pathology.

## CONFLICT OF INTEREST

The authors declare no conflict of interest.
